# Role of GALNT4 in protecting against cardiac hypertrophy through ASK1 signaling pathway

**DOI:** 10.1038/s41419-021-04222-5

**Published:** 2021-10-22

**Authors:** Bin-Bin Zhang, Lu Gao, Qin Yang, Yuan Liu, Xiao-Yue Yu, Ji-Hong Shen, Wen-Cai Zhang, Zhan-Ying Han, Shao-Ze Chen, Sen Guo

**Affiliations:** 1 Department of Cardiology, The First Affiliated Hospital of Zhengzhou University, No.1 Jianshe East Road, Zhengzhou, China; 2 Department of Cardiology, Huanggang Central Hospital, Huanggang, China; 3 Huanggang Institute of Translational Medicine, Huanggang, China; 4 Department of Electrocardiogram, The Second Affiliated Hospital of Zhengzhou University, No.2 Jingba Road, Zhengzhou, China

**Keywords:** Protein-protein interaction networks, Heart failure

## Abstract

Pathological myocardial hypertrophy is regulated by multiple pathways. However, its underlying pathogenesis has not been fully explored. The goal of this work was to elucidate the function of polypeptide N-acetylgalactosaminyltransferase 4 (GALNT4) in myocardial hypertrophy and its underlying mechanism of action. We illustrated that GALNT4 was upregulated in the models of hypertrophy. Two cardiac hypertrophy models were established through partial transection of the aorta in GALNT4-knockout (GALNT4-KO) mice and adeno-associated virus 9-GALNT4 (AAV9-GALNT4) mice. The GALNT4-KO mice demonstrated accelerated cardiac hypertrophy, dysfunction, and fibrosis, whereas the opposite phenotype was observed in AAV9-GALNT4 mice. Similarly, GALNT4 overexpression mitigated the degree of phenylephrine-induced cardiomyocyte hypertrophy in vitro whereas GALNT4 knockdown aggravated the hypertrophy. In terms of mechanism, GALNT4 deficiency increased the phosphorylation and activation of ASK1 and its downstream targets (JNK and p38), whereas GALNT4 overexpression inhibited activation of the ASK1 pathway. Furthermore, we demonstrated that GALNT4 can directly bind to ASK1 inhibiting its N-terminally mediated dimerization and the subsequent phosphorylation of ASK1. Finally, an ASK1 inhibitor (iASK1) was able to reverse the effects of GALNT4 in vitro. In summary, GALNT4 may serve as a new regulatory factor and therapeutic target by blocking the activation of the ASK1 signaling cascade.

## Introduction

Cardiac hypertrophy can accelerate the process of heart failure (HF), leading to significant impacts on morbidity and mortality. Both the incidence and 5-year case fatality rates of HF are expected to increase by nearly 50% in the next decade [1]. HF can be caused by a variety of diseases, such as aortic valve stenosis and arrhythmia, while cardiac hypertrophy is a resultant adaptation to these chronic stimuli [2]. Both physiological and pathological hypertrophy are caused by a compensatory increase in protein and the enlargement of myocardial tissue, but their phenotypes, mechanisms, and treatments are significantly different. Physiological cardiac hypertrophy can maintain normal heart function, while pathological cardiac hypertrophy can cause many serious cardiovascular events, such as cardiac arrest and even death [3]. Management strategies for people with heart failure caused by pathological cardiac hypertrophy include drug and non-drug treatments, which together improve the quality of life, but mortality remains very high [4]. The mechanisms underlying cardiac hypertrophy and HF are still not fully understood [5]. Therefore, further study is urgently required to facilitate the development of new treatments for HF.

PpGalNAc-T, family member polypeptide N-acetylgalactosaminyltransferase 4 (GALNT4), also known as GalNAc-T4, is a transmembrane protein encoded by an exon [6, 7]. GALNT4 participates in post-translational modifications of target proteins through O-GalNAc glycosylation, and is involved in invasion, proliferation, and other functions [8–10]. Previous studies have found that GALNT4, as a downstream molecule of miR-4262, is involved in the tumor proliferation and apoptosis in colon cancer [9]. In addition, in liver cancer, GALNT4 has been shown to modulate the activation of EGFR, and a decrease in the miR-9/GALNT4 axis has also been shown to promote the malignant transformation of liver cells [10]. A recent study also found that GALNT4 expression levels in blood of patients with acute coronary syndrome (ACS) are significantly higher than in control subjects [11]. However, whether GALNT4 is a regulatory factor in pathological cardiac hypertrophy, and its possible underlying mechanism remains unknown.

Therefore, our work aimed to investigate the function of GALNT4 in cardiac hypertrophy and its underlying mechanism of action. The results suggested that GALNT4 was highly expressed in mouse and cardiomyocyte models of pathological cardiac hypertrophy. We then proved that GALNT4 can negatively regulate cardiac remodeling by GALNT4 loss and gain of function. Furthermore, we demonstrated that GALNT4 could directly bind to ASK1, inhibiting the dimerization and phosphorylation of ASK1 and mitigating cardiac hypertrophy by inhibiting the ASK1 axis.

## Materials and methods

### Animal models

All relevant experimental procedures were conducted in accordance with the Guide for the Care and Use of Laboratory Animals, and approved by the Animal Care and Use Committee of the First Affiliated Hospital of Zhengzhou University.

### Production of GALNT4-KO mice

The CRISPR online design tool (http://chopchop.cbu.uib.no/) was used to predict the guiding sequence as follows: guideRNA1 target site: 5′-GCCTGAGTGAGCCAGGCGCGATGGG-3′, guideRNA2 target site: 5′-TTGGGGCACGGTGACTTTCCGGAGG-3′, which resulted in a 2 kb deletion in total. The GALNT4-sgRNA expression vector was constructed on the BsaI restriction site of pUC57-sgRNA (Addgene, 51132). The products of the Cas9 expression vector pST1374-Cas9 (Addgene 44758) and the sgRNA expression vector were mixed and microinjected into C57BL/6J zygotes with the FemtoJet 5247 microinjection system. F0 generation mice were obtained after 19–21 days, and were identified by PCR and sequencing DNA was extracted from the ear tissue of 2-week-old mice and assessed using the primers below: GALNT4-check F1: 5′-AAGGTCACAAAGCTGCCATC-3′, GALNT4-check R1: 5′- GGAGGTGCAATCGACCTTTA-3′.

### Production of AAV9-GALNT4 mice

AAV9-GALNT4 and control mice were constructed according to previously described methods [12]. Briefly, GALNT4 was composed of cytomegalovirus (CMV) promoter and human GALNT4 sequence, and GALNT4 was transported into the pAAV vector via the NheI and EcoRI site, and the recombinant plasmid was then transfected into *E. coli* DH5a. Next, the recombinant plasmids, Helper, and pAAV RC were cotransfected into AAV-293 cells. The recombinant plasmid was purified using a CsCl_2_ after three days of transfection. The titrations of AAV9-GALNT4 and AAV9 control were detected by RT-PCR. Mice were injected with 7.5 × 10^11^ (viral genomes) VG of AAV9-GALNT4 or control subjects via the tail vein two weeks before transverse aortic constriction (TAC) surgery.

### Animal surgery

Male mice (25.5–27 g, 9–11 weeks old) were anesthetized with sodium pentobarbital (90 mg/kg). After the toe pinch reflex disappeared, the mice were kept at 37 °C during the experiment. The thymus was excised and the aortic arch exposed. A 7–0 silk thread was passed through the aortic arch, and a 26-G needle was placed parallel to the aortic arch. The blood vessel was ligated with the needle, which was then withdrawn, and the mice subsequently placed in a 37 °C incubator. Mice were randomly assigned to each group.

### Echocardiography assessment

A small animal ultrasound imaging system (VEVO2100, FUJIFILM VISUALSONICS, Canada) and a 30 MHz (MS400) linear-array ultrasound transducer were used to examine the mice. The size of the left ventricle (LV) cavity, LV wall thickness, LV end-diastolic diameter, LV end-systolic diameter, ejection fraction (EF%), and fractional shortening (FS%) were detected at the mid-papillary muscle level in each group, through echocardiography in the M-mode.

### Animal samples

Four weeks after the TAC operation, the weight of the mice was measured and recorded. The hearts were excised and quickly placed in a 10% KCl solution. The dry weight of the hearts was obtained prior to fixing in liquid nitrogen or 10% formalin. The lung weights and tibial lengths were also measured.

### Histological examination

The hearts were fixed for 48 h and serial cross-sections sliced continuously at a thickness of 5 μm. Hematoxylin (Servicebio, G1004) and eosin (Baso, BA-4024) (H & E) staining and Sirius red picrate (Hedebiotechnology Co., Ltd., 26357-02) (PSR) staining were used to aid microscopic measurements of the cell-surface area (CSA) and the collagen content of myocardial cells, respectively, by using Image Pro Plus 6.0.

### Western blotting

Lysates of the left ventricular tissue or the heart were prepared by the addition of RIPA buffer (720 μL of RIPA buffer, 20 μL of phenylmethylsulfonyl fluoride, 100 μL of complete protease inhibitor cocktail, 100 μL of Phos-stop, 50 μL of NaF and 10 μL of Na3 VO4 in a final volume of 1 mL). After centrifugation, the resulting supernatant was assumed to represent total tissue protein. Protein levels were quantified using a BCA protein Kit (Pierce). Identical amounts of protein were separated via 10% SDS-PAGE gel electrophoresis, and the separated proteins were electrophoretically transferred to a 0.45 μm polyvinylidene difluoride (PVDF) membrane (IPVH00010, Millipore). The membranes were treated with 5% skim milk powder for 1 h at room temperature (approximately 25 °C), then washed three times with TBST, prior to incubation overnight at 4 °C with the appropriate antibodies. Subsequently, the membranes were washed again with TBST and then incubated with the appropriate secondary antibody (Jackson Immune Research) for 1 h at room temperature. Immunoreactive proteins were visualized using a Bio-Rad ChemiDoc XRS+ system (1705062, Bio-Rad). GADPH was used to normalize protein levels. The antibodies in this work are shown in Supplementary Table 1.

### Quantitative real-time (RT) PCR

Total RNA was extracted using TRIzol reagent (15596-026, Invitrogen). The total RNA was reverse transcribed into cDNA using the Transcriptor First Strand cDNA Synthesis Kit (04896866001, Roche), with the mRNA levels of GAPDH being used for normalization. The sequence of the primers are listed in Supplementary Table 2.

### Construction of the adenoviral vectors used in cell culture

The GALNT4 gene was cloned into a replication defect adenovirus vector controlled by the CMV promoter and used to overexpress GALNT4. The replication deficient adenovirus vector carrying a short hairpin RNA targeting GALNT4 was used to reduce the expression levels of GALNT4, while the AdshRNA adenovirus was used as a control. Adenovirus infections were carried out an MOI of 50 for 24 h and then identified. The primer sequences used for analysis were: AdGALNT4-mouse-F: 5′-GGCTAGCGATATCGGATCCGCCACCATGGCCGTGAGGTGGACCTG-3′; AdGALNT4-mouse-R: 5′-CGTCCTTGTAATCACTAGTTTTCTCAAACCTCCAGAGCTGG-3′; AdshGALNT4-rat-F, 5′-CCGGTGAGTGTAACACTGGTTGGTTCTCGAGAACCAACCAGTGTTACACTCATTTTTG-3′; AdshGALNT4-rat-R, 5′-AATTCAAAAATGAGTGTAACACTGGTTGGTTCTCGAGAACCAACCAGTGTTACACTCA-3′.

### Cell culture and transduction

1-2 days Sprague-Dawley rats were selected; the major blood vessels were removed from the heart after which the heart tissue was cut into 1–2 cm^3^ pieces. Trypsin (0.125%) was added for tissue digestion and the resulting NRCMs were cultured in DMEM/F12 (C113305, Gibco) medium (10% FCS, 1% PS, and 0.1 mM 5-Brdu) for 24 h, after which myocardial cells were infected with indicated adenovirus. After 6 h of infection, the serum-free medium was replaced and the cells incubated for 12 h, after which PE (50 μM) or an inhibitor of ASK1 GS4997 (iASK1, 1148428-04-3, Selleck, 80 μM) was added for 24 h. PBS or DMSO were added in the control cells. Cell cultures were maintained at 37 °C and 5% CO_2_.

### Immunofluorescence staining

After 24 h of culture, the cardiomyocytes were fixed with 4% formaldehyde for 30 min, permeabilized with 0.2% Triton X-100, and the fixed cells blocked with 8% BSA. They were then incubated with an α-actinin antibody (05-384, Merck Millipore, 1:100 dilution), followed by the appropriate secondary antibody (A21202, Invitrogen, 1:200 dilution), and DAPI (0100-20, Southern Biotech, nuclear stain). The surface area of the cardiomyocytes was calculated using Image Pro Plus 6.0.

### Immunoprecipitation

293T cells were transfected with the appropriate plasmids. After 24 h of co-transfection, the cells were lysed with IP lysis buffer (20 mM Tris-HCl, pH 7.4; 150 mM NaCl; 1 mM EDTA; 1% NP-40; cocktail). The lysates were centrifuged at high speed at 4 °C, then the supernatant was incubated with protein G agarose beads and the labeled antibody at 4 °C overnight. After centrifugation (3000 rpm at 4 °C) the beads were washed three times each with a buffer containing 300 mM and 150 mM NaCl. After which the beads were incubated with 2× SDS loading buffer, boiled at 95 °C for 5–10 min, and then analyzed by western blotting (WB).

### GST pull-down assay

GST-HA-ASK1, FLAG-GALNT4, GST-HA-GALNT4, and FLAG-ASK1 were overexpressed in eukaryotic cells, after which the cells were lysed with lysis buffer (50 mM Na_2_HPO_4_, pH 8.0; 300 mM NaCl; 1% TritonX-100; cocktail). GST-HA-GALNT4 or GST-HA-ASK1 were purified using GST beads. GST-HA-GALNT4 and FLAG-ASK1 or GST-HA-ASK1 and FLAG-GALNT4 were mixed and incubated overnight at 4 °C. The beads were washed three time with buffer, resuspended in 2X SDS loading buffer, boiled at 95 °C for 5–10 min, and then analyzed by WB.

### Statistical analysis

All data was presented as the mean ± SD. A two-tailed *t*-test was used to compare the data between the two groups. A two-way analysis of variance (ANOVA) without repeated measures was applied for multiple comparisons with two independent variables. After confirming that an interaction effect was statistically significant, a one-way ANOVA was used to compare the data between multiple groups, and the Bonferroni test (assuming homogeneous variance) or Tamhane’s T2 test (assuming uneven variance) was used for corrections. SPSS version 25.0 was used to conduct the statistical analyses. Statistical significance was set at *p* < 0.05.

## Results

### GALNT4 is upregulated in experimental models of hypertrophy

**Fig. 1:**
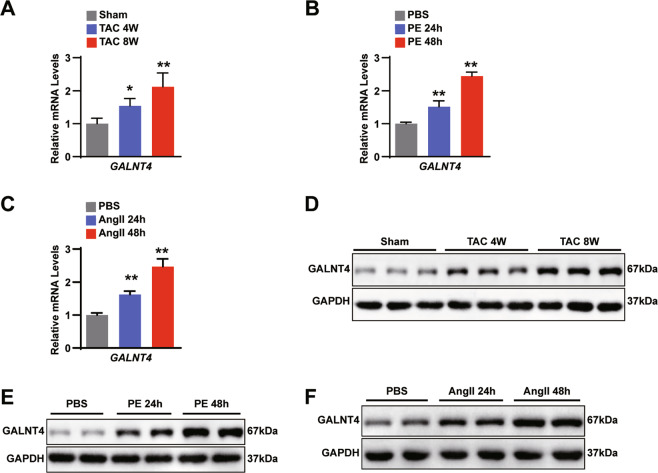
GALNT4 is upregulated in experimental models of hypertrophy. **A** Real-time PCR of GALNT4 expression in TAC group and sham group after 4 or 8 weeks. **B**, **C** The mRNA level of GALNT4 in NRCMs in the PE group and the Ang II group, respectively after 24 or 48 h. **D**–**F** Western blot of GALNT4 expression in every group. *p < 0.05 vs. sham or PBS, **p < 0.01 vs. sham or PBS.

To explore the role of GALNT4, we first measured its levels in cell and animal models of cardiac hypertrophy. In Fig. 1A, a qPCR analysis showed that GALNT4 mRNA levels were upregulated 4 or 8 weeks following TAC surgery. Furthermore, in vitro the mRNA level of GALNT4 increased at 24 or 48 h after phenylephrine (PE) and angiotensin II (Ang II) treatment (Fig. 1B, C). Consistent with the results of qPCR, WB analyses also showed that the protein level of GALNT4 increased significantly in both mice and NRCMs (Fig. 1D–F). The above findings suggested that GALNT4 may play a role in cardiomyocyte hypertrophy.

### GALNT4 suppresses cardiomyocytes hypertrophy in vitro

**Fig. 2:**
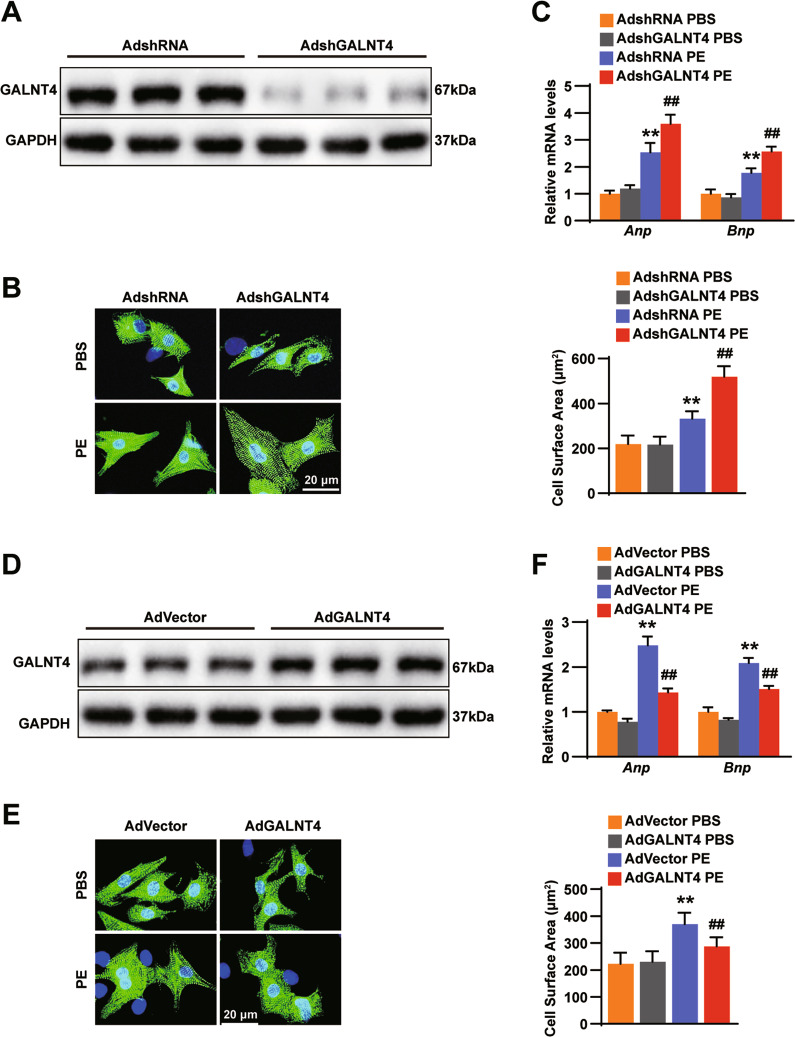
GALNT4 suppresses PE-induced cardiomyocytes hypertrophy. **A** The overexpression of GALNT4 was verified by western blotting of cardiomyocytes (*n* = 3 independent experiments). **B** Immunofluorescence staining images of NRCMS treated with AdshGALNT4 or AdshRNA after treatment with PE or PBS in each group (*n* = 30-40 cells). **C** Changes in cardiac hypertrophy markers were assessed after treatment with PE or PBS by real-time PCR (*n* = 3 independent experiments). **D** GALNT4 protein levels after transduction with AdGALNT4 and control (*n* = 3 independent experiments). **E** Immunofluorescence staining images of cells infected with AdGALNT4 or AdVector and treated with PE or PBS (*n* = 30-40 cells). **F** The level of cardiac hypertrophy markers in NCRMs was detected by real-time PCR (*n* = 3 independent experiments). **p < 0.01 vs. AdshRNA PBS or AdVector PBS, ^**##**^*p* < 0.01 vs. AdshRNA PE or AdVector PE.

An increase in myocardial cell area and an increase in corresponding markers are the main changes in myocardial hypertrophy [13]. GALNT4 expression was suppressed following infection of cardiomyocytes with an adenoviral short hairpin RNA targeting GALNT4 (AdshGALNT4) (Fig. 2A). In Fig. 2B, C, PE could induce the process of cardiomyocytes hypertrophy. Compared with control cells, GALNT4-knockdown NRCMs displayed a significant increase in their CSA and expression of the hypertrophic markers ANP and BNP. GALNT4 levels were increased following infection with adenoviral GALNT4 (AdGALNT4) (Fig. 2D). Infection with AdGALNT4 significantly inhibited the cardiomyocyte hypertrophy triggered by PE (Fig. 2E, F). These results showed that upregulation of GALNT4 levels protects against cardiomyocyte hypertrophy and conversely downregulation of GALNT4 promotes the development of cardiomyocyte hypertrophy.

### GALNT4 deficiency accelerates the cardiac hypertrophy induced by TAC

**Fig. 3:**
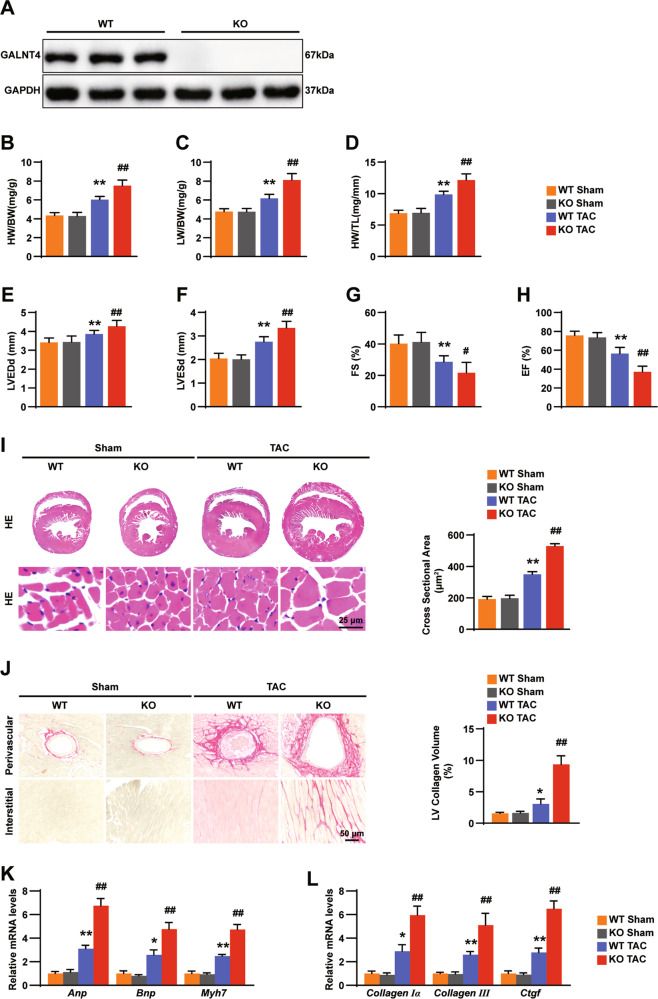
GALNT4 deficiency accelerates cardiac hypertrophy in vitro. **A** The levels of GALNT4 in GALNT4-KO mice and WT mice were analyzed by western blotting. **B**–**D** The ratio of HW/ BW, LW/BW, and HW/TL in each group (*n* = 10 mice per group). **E**–**H** LVEDd, LVESd, EF%, and FS% were analyzed in every group (*n* = 10 mice per group). **I** H&E staining images (*n* = 6 mice per group) and heart tissue CSA (*n* = 120 cells in each group) in the GALNT4-KO group and WT control. **J** Images of PSR staining (left, *n* = 6 mice per group) and percentage of LV collagen volume in the indicated group. **L**, **M** The expression of hypertrophic and fibrotic markers were analyzed by qPCR (*n* = 4 mice per group). *p < 0.05 vs. WT sham, **p < 0.01 vs. WT sham, ^**#**^*p* < 0.05 vs. WT TAC, ^**##**^*p* < 0.01 vs. WT TAC.

GALNT4 global knockout mice were used to confirm the function of GALNT4 in the hypertrophic model treated by TAC surgery. The GALNT4 protein was confirmed to not be expressed in the GALNT4 global KO mice (Fig. 3A). TAC or sham operation was performed on GALNT4-KO and WT mice. Four weeks later, we found no differences in cardiac structure and function between WT sham group and GALNT4-KO sham group. However, in the TAC surgery group, the HW/BW, LW/BW, HW/TL ratios, as well as LVEDd and LVESd in the GALNT4-KO mice were significantly higher than those in WT mice (Fig. 3B–F). Furthermore, in terms of heart function, both EF% and FS% in GALNT4-KO mice were lower than those in WT mice (Fig. 3G, H). Following H&E and PSR staining, GALNT4-KO mice showed a larger cardiomyocyte area (Fig. 3I) and more obvious myocardial fibrosis (Fig. 3J). Furthermore, markers of myocardial hypertrophy and fibrosis were higher in GALNT4-KO mice than those in WT mice (Fig. 3K, L). From the above results, we concluded that the absence of GALNT4 aggravates the level of myocardial hypertrophy.

### Cardiomyocyte-specific overexpression of GALNT4 attenuates the cardiac hypertrophy induced by TAC surgery

**Fig. 4:**
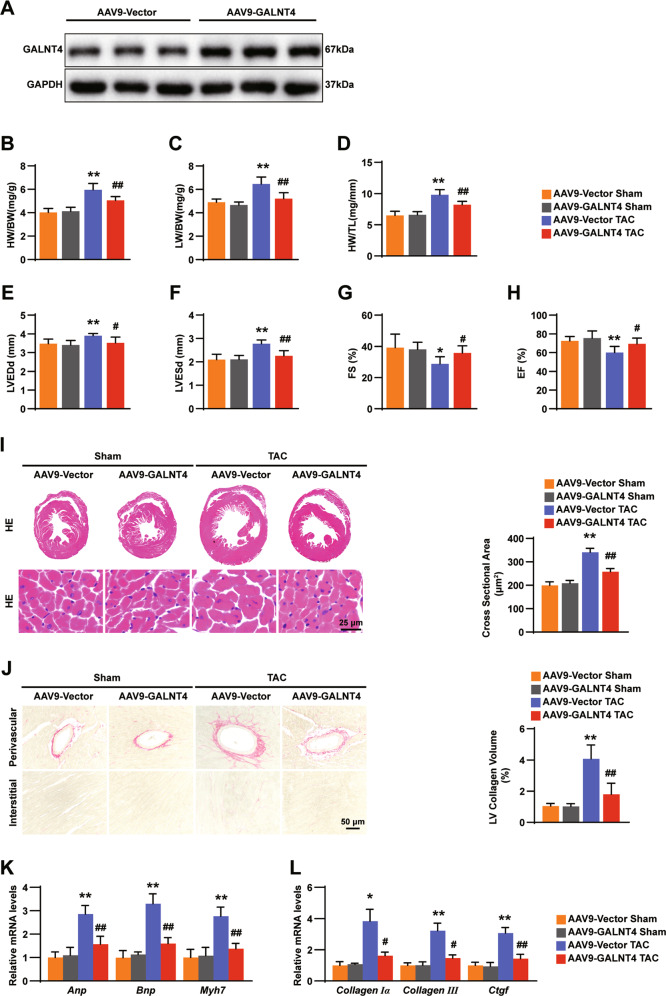
Cardiomyocyte-specific overexpression of GALNT4 attenuates the cardiac hypertrophy induced by TAC surgery. **A** The levels of GALNT4 in AAV9–GALNT4, AAV9-Vector mice were analyzed by western blotting. **B**–**D** The ratio of HW/ BW, LW/BW, and HW/TL in the AAV9-GALNT4 group and sham group (*n* = 10 mice per group). **E**–**H** LVEDd, LVESd, EF%, and FS% were analyzed in each group (*n* = 10 mice per group). **I** H&E staining images (*n* = 6 mice per group) and heart CSA (*n* = 120 cells in each group) in AAV9–GALNT4 and control mice. **J** Images of PSR staining (left, *n* = 6 mice per group) and percentage of LV collagen volume in the indicated group. **L**, **M** The levels of markers of hypertrophy and fibrosis were analyzed by qPCR (*n* = 4 mice per group). *p < 0.05 vs. AAV9-Vector sham, **p < 0.01 vs. AAV9-Vector sham, ^**#**^*p* < 0.05 vs. AAV9-Vector TAC, ^**##**^*p* < 0.01 vs. AAV9-Vector TAC.

The AAV9 gene transfer method was chosen, owing to its proven chemotaxis in cardiomyocytes [14]. GALNT4 protein was confirmed to be overexpressed in AAV9-GALNT4 mice (Fig. 4A). The HW/BW, LW/BW, and HW/TL ratios in the heart were decreased in the AAV9–GALNT4 group compared to those in the AAV9-Vector group after 4 weeks of TAC, while there was no difference in the sham operation (Fig. 4B–D). Moreover, AAV9–GALNT4 prevented heart enlargement, hypertrophy, and dysfunction in the TAC surgery mice (Fig. 4E–H). Compared to AAV9-Vector mice, the CSA plus interstitial and perivascular fibrosis of myocytes from the AAV9–AALNT4 mice were significantly reduced, as revealed by H&E and PSR staining (Fig. 4I, J). As mentioned above, AAV9–GALNT4 mice showed a significant decrease in the expression of several markers of hypertrophy and fibrosis (Fig. 4K, L). Overall, the above findings demonstrated that overexpression of GALNT4 protects against hypertrophy, fibrosis, and decreased cardiac function triggered by TAC.

### GALNT4 regulates pathological myocardial hypertrophy through the ASK1-JNK/p38 signaling pathway

**Fig. 5:**
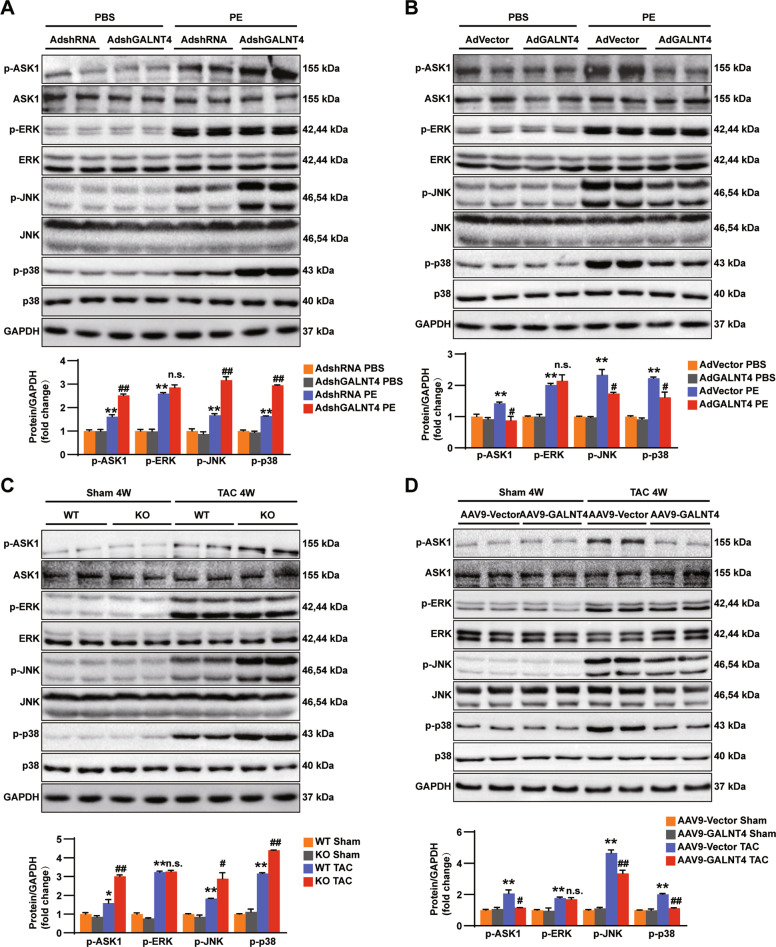
GALNT4 inhibits the ASK1 signaling pathway. **A**, **B** The expression of phosphorylated and total ASK1 and its downstream molecules in cells treated with the indicated adenovirus. **A**, **B** **p < 0.01 vs. AdshRNA PBS or AdVector PBS, ^**#**^*p* < 0.05 vs. AdshRNA PE or AdVector PE, ^**##**^*p* < 0.01 vs. AdshRNA PE or AdVector PE, n.s. indicates no significance. **C**, **D** Phosphorylated and total ASK1 and its downstream molecules in mouse hearts in each group subjected to TAC or sham surgery. **C**, **D** *p < 0.05 vs. WT sham or AAV9-Vector sham, **p < 0.01 vs. WT sham or AAV9-Vector sham, ^**#**^*p* < 0.05 vs. WT TAC or AAV9-Vector TAC, ^**##**^*p* < 0.01 vs. WT TAC or AAV9-Vector TAC. n.s. indicates no significance.

We next investigated the signaling pathway by which GALNT4 regulates cardiac hypertrophy. Previous studies have indicated that MAPK is the most common pathway regulating cardiac hypertrophy [3, 15]. Therefore, we examined the activation levels of ASK1 and its downstream molecules in mouse hearts and NRCMs overexpressing or underexpressing GALNT4. Notably, compared with control cells, AdshGALNT4 clearly promoted the phosphorylation of ASK1, JNK, and p38 in PE-treated cardiomyocytes; however, the activation of ERK was not changed by the knockdown of GALNT4 (Fig. 5A). Overexpression of GALNT4 significantly inhibited the phosphorylation of these molecules, with the exception of ERK (Fig. 5B). A similar result was obtained from the in vivo study (Fig. 5C, D). Based on these data, we concluded that GALNT4 modulates cardiac hypertrophy and cardiac fibrosis through the ASK1-JNK/p38 signaling pathway.

### GALNT4 regulates apoptosis and directly binds to ASK1 to inhibit its oligomerization

**Fig. 6:**
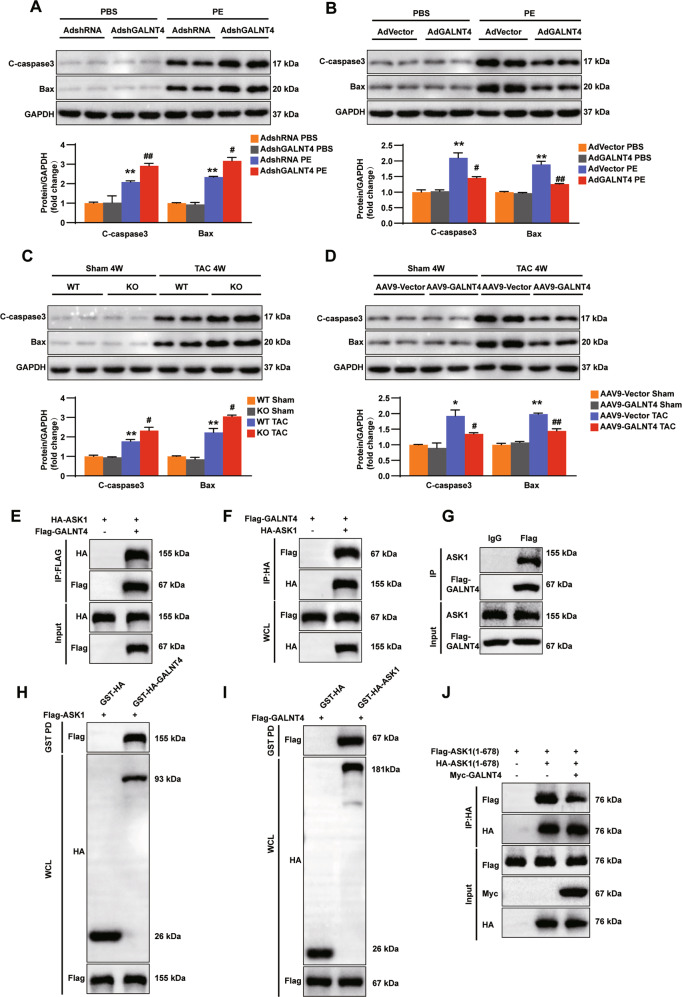
GALNT4 can regulate apoptosis and directly binds to ASK1 to inhibit its oligomerization. **A** Knockdown of GALNT4 increases the expression of apoptotic proteins in vivo. **B** The overexpression of GALNT4 inhibits cardiomyocytes apoptosis. **A**, **B** **p < 0.01 vs. AdshRNA PBS or AdVector PBS, ^**#**^*p* < 0.05 vs. AdshRNA PE or AdVector PE, ^**##**^*p* < 0.01 vs. AdshRNA PE or AdVector PE. **C**, **D** The expression of Bax and C-caspase 3 in vitro. For C-D, *p < 0.05 vs. WT sham or AAV9-Vector sham, **p < 0.01 vs. WT sham or AAV9-Vector sham, ^**#**^*p* < 0.05 vs. WT TAC or AAV9-Vector TAC, ^**##**^*p* < 0.01 vs. WT TAC or AAV9-Vector TAC. **E**, **F** The interaction between GALNT4 and ASK1 in 293T cells. **G** The interaction between GALNT4 and ASK1 in NRCMs. **H**, **I** A GST pull down assay was used to verify the direct combination between GALNT4 and ASK1. **J** The effect of GALNT4 on the dimerization of ASK1 (1–678) in 293T cells.

As mentioned above, ASK1 can regulate cell apoptosis. The question as to whether altered GALNT4 expression levels can regulate apoptosis in vivo or in vitro following PE treatment or TAC surgery, respectively is unknown. To address this, we assessed the expression degree of the apoptosis related proteins in vivo and vitro. We found that knockdown of GALNT4 increased the expression of Bax and C-caspase3 in vivo (Fig. 6A), whereas overexpression of GALNT4 reduced the expression of these proteins (Fig. 6B). Furthermore, similar results were seen in vivo following TAC surgery (Fig. 6C, D). Collectively, these results indicated that GALNT4 participates in regulating cardiomyocyte survival and apoptosis.

Taken together the data strongly suggest that GALNT4 regulates cardiac hypertrophy via the MAPK axis. However, the specific mechanism by which GALNT4 regulates the ASK1-JNK/p38 axis is unknown. To further investigate this issue, Flag-GALNT4 and HA-ASK1 were expressed in 293T cells. Significantly, both co-immunoprecipitation (co-IP) of GALNT4 using an anti-FLAG antibody and co-IP of ASK1 using an anti-HA antibody showed an interaction between GALNT4 and ASK1 in 293T cells (Fig. 6E, F). Furthermore, the interaction between GALNT4 and ASK1 was seen in NRCMs (Fig. 6G). In addition, a GST pulldown was used to verify the direct combination between GALNT4 and ASK1 (Fig. 6H, I). Dimerization of ASK1 is crucial for the phosphorylation and activation of ASK1 [16]. We constructed a series of peptide fragments to explore the interaction between GALNT4 and ASK1. The results demonstrated that GALNT4 can inhibit the N-terminal dimerization of ASK1 (Fig. 6J). The above results demonstrated that GALNT4 can directly bind to ASK1 and regulate its N-terminal dimerization.

### Inhibiting ASK1 is a crucial step in regulating cardiac hypertrophy through GALNT4

**Fig. 7:**
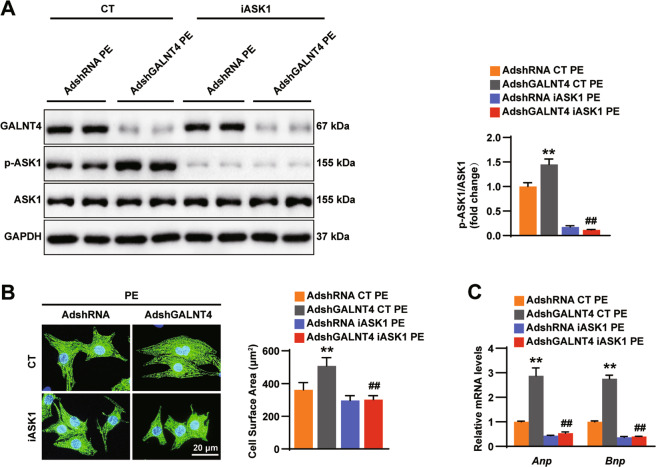
Inhibiting ASK1 is the key step by which GALNT4 regulates hypertrophy. **A** The protein levels of GALNT4, pASK1, and ASK1 in NRCMS infected with the indicated adenovirus and treated with iASK1 or not after treatment with PE. **B** Images of NRCMS in each group and the CSA (*n* = 30-40 cells). **C** The mRNA expression levels of ANP and BNP were examined by RT qPCR. *n* = 3 independent experiments. **p < 0.01 vs. AdshRNA, ^**##**^*p* < 0.01 vs. AdshGALNT4.

An inhibitor of ASK1 (iASK1) was used to validate whether inhibiting ASK1 is necessary for GALNT4 to protect against cardiomyocyte hypertrophy (Fig. 7A). After treatment with PE, in comparison to the AdshRNA NRCMs, AdshGALNT4 expressing NRCMs displayed obvious hypertrophy based on the level of pASK1. However, treatment with iASK1 partially attenuated the effects of AdshGALNT4 (Fig. 7B, C). All of these data strongly suggested that GALNT4 directly binds to ASK1, inhibits its oligomerization, decreasing signaling through the ASK1-JNK/p38 cascade, thereby regulating cardiac remodeling.

## Discussion

As the end stage of several diseases, HF has serious consequences on morbidity and mortality; however, its mechanism has not yet been fully explored and understood [17]. Our study found that GALNT4 could protect against pathological hypertrophy, yet it does not function under normal physiological conditions. We also found that GALNT4 could bind to ASK1 and inhibit its oligomerization and phosphorylation to regulate cardiac hypertrophy.

The ppGalNAc-T family is highly conserved in eukaryotes, and members of this gene family exist from *Toxoplasma gondii* to mammals [18]. Different ppGalNAc-T enzymes have a relative conservation of 40–60% in amino acid sequence, and there is a certain degree of redundancy in function [7]. GALNT4 participates in post-translational modification of proteins as well as in many biological processes, including signal pathway activation, protein transport, and cell migration [9]. A recent study has found that PSGL-1-positive monocytes and GALNT4 levels in the blood of patients with ACS are higher than those control subjects, and further studies have confirmed that GALNT4 participates in the synthesis of PSGL-1, activates the Akt/mTOR pathway, and increases its phosphorylation level [11].

Here, we summarize the function of GALNT4 and its regulation of pathological cardiac hypertrophy. Our study indicated that GALNT4 is upregulated during the pathological process of cardiac hypertrophy. Knockout or knockdown of the *GALNT4* gene in mice enhanced cardiac remodeling, while its overexpression mitigated remodeling. Similar results were obtained in vitro. Furthermore, we found that ASK1 is the downstream core target of GALNT4 that regulates cardiac hypertrophy. Our study indicated that GALNT4 regulates the ASK1 by significantly inhibiting its N-terminal dimerization and the subsequent phosphorylation of ASK1. ASK1 is a MAPKKK, which has been shown to be an activator of its downstream targets (JNK and p38) [19, 20]. GALNT4 directly interacts with ASK1 to reduce the phosphorylation of the ASK1 pathway, thereby regulating cardiac hypertrophy.

Pathological myocardial hypertrophy involves many signaling pathways, among which the most studied are the MAPK, NFAT, STAT, PKC, PI3K/PKB, NF-κB, and Wnt pathways [3, 21–23]. As a part of the MAPK signaling system, ASK1 is a MAPKKK that has been identified as being crucial in the processes of apoptosis, oxidative stress, and myocardial remodeling. When ASK1 is activated, it can promote cell apoptosis and oxidative stress, leading to tissue damage [24, 25]. ASK1 has recently received more attention in the process of cardiovascular diseases [25, 26]. Many studies have confirmed that ASK1 is indeed a kinase that is involved in heart failure and cardiac hypertrophy [27]. Huang et al. have also reported that an increase in ASK1 expression is related to increased cardiomyocyte apoptosis, and the use of highly selective ASK1 inhibitors improves the cardiac phenotype and ventricular remodeling, relieves oxidative stress, suggesting that inhibiting ASK1 is a potential treatment for heart failure [25]. ASK1 is regulated by C1B1, ROS, TNF receptor-related factors (TRAFs), translocase of the inner membrane 50 (TIM50), and other factors [24, 28–30]. Tang et al. found that TIM50 is related to the regulation of myocardial hypertrophy by regulating the ASK1-JNK/p38 signaling pathway [30]. Previous studies have shown that ASK1 promotes cardiomyocyte death one week after TAC surgery, due to an up-regulation of Bax [31]. There is also another study showing that isoproterenol (ISO) can induce cardiac apoptosis [32]. In our study, we also found that GALNT4 inhibits cardiomyocyte apoptosis in cardiac hypertrophic models.

The activation of ASK1 is controlled by its oligomerization, as well as posttranslational modifications such as phosphorylation, ubiquitination, and methylation [33, 34]. ASK1 forms a complex including other molecules in resting cells, and these protein forms a homo-oligomer through binding with the C-terminal of ASK1; however, this is not sufficient to cause the activation of ASK1. On the basis of C-terminal binding, N-terminal dimerization is the key to activation [16]. Importantly, our study indicated that GALNT4 inhibits the development of cardiac remodeling by directly binding to ASK1 and inhibiting its oligomerization and subsequent phosphorylation. The JNK/p38 axis is a significant part of the MAPK signaling pathway [35]. In rat models of pulmonary hypertension as well as in vitro, inhibiting the ASK1-JNK/p38 axis has been shown to reduce pathological myocardial remodeling [36]. Collectively, GALNT4 inhibits the JNK/p38 cascade by negatively regulating the activation of ASK1, thereby regulating cardiac hypertrophy.

ERK is also a significant part of the MAPK pathway and is regulated by ASK1 [37]. Many studies have shown that ERK is related to pathological cardiac hypertrophy [38, 39]. However, we did not find any changes of ERK phosphorylation (Fig. 5). It is possible that ERK is also regulated by other important molecules and this requires further research.

This study has several limitations. First, global GALNT4 knockout mice were used in our study instead of the cardiomyocyte-specific type. Second, although we have demonstrated that GALNT4 could directly bind to ASK1 and inhibit its oligomerization and phosphorylation, the detailed molecular relationship between GALNT4 and activation of ASK1 requires further clarification.

In conclusion, we have explored the role of GALNT4 on the inhibition of cardiac hypertrophy through the ASK1 signaling pathway. Our work demonstrates that GALNT4 is a negative regulatory factor of myocardial hypertrophy and that it does not play a role under physiological conditions. Overexpression of GALNT4 mitigates cardiac hypertrophy and cardiac fibrosis, suggesting that GALNT4 may be a promising target for the treatment of cardiac hypertrophy.

## Supplementary information

Supplementary Data

## Data Availability

The datasets generated and/or analyzed during the current study are available from the corresponding author on reasonable request.
