# Supplementary material for: Role of GALNT4 in protecting against cardiac hypertrophy through ASK1 signaling pathway
Source: Cell Death Dis. 2021 Oct 22;12(11):980. doi: 10.1038/s41419-021-04222-5 (PMC8531281; doi:10.1038/s41419-021-04222-5)
Supplement: Supplementary file 1 — Supplementary Data [file 41419_2021_4222_MOESM1_ESM.docx]

**Supplementary Data**

**Role of GALTN4 in protecting against cardiac hypertrophy through ASK1 signaling pathway**

Bin-Bin Zhang^1*^, Lu Gao^1*^, Qin Yang^2,3*^, Yuan Liu^1^, Xiao-Yue Yu^1^, Ji-Hong Shen^4^, Wen-Cai Zhang^1^, Zhan-Ying Han^1#^, Shao-Ze Chen^2,3#^ and Sen Guo^1#^

Article Information:

^1^Department of Cardiology, The First Affiliated Hospital of Zhengzhou University,

No.1 Jianshe East Road, Zhengzhou, China;

^2^Department of Cardiology, Huanggang Central Hospital, Huanggang, China;

^3^Huanggang Institute of Translational Medicine, Huanggang, China;

^4^Department of Electrocardiogram, The Second Affiliated Hospital of Zhengzhou

University, No.2 Jingba Road, Zhengzhou, China

*These authors contributed equally to this work

**Corresponding authors**

**Sen Guo, Ph.D.**

Department of Cardiology, the First Affiliated Hospital of Zhengzhou University,

Zhengzhou University.

Tel: 86-0371-67967622;

Fax: 86-0371-67967626;

E-mail: [fccguos@zzu.edu.cn](mailto:fccguos@zzu.edu.cn);

**Shao-Ze Chen,** **Ph.D.**

Department of Cardiology, Huanggang Central Hospital, Huanggang, China;

Huanggang Institute of Translational Medicine, Huanggang, China;

Tel: 86-0713-8381191;

Fax: 86-0713-8381191;

E-mail: [chenshaoze@hgyy.org.cn](mailto:chenshaoze@hgyy.org.cn)

**Zhan-Ying Han, M.D., Ph.D.**

Professor

Department of Cardiology, the First Affiliated Hospital of Zhengzhou University,

Zhengzhou University.

No.1 Jianshe East Road, Zhengzhou, Henan 450052, China;

Tel: 86-0371-67967622;

Fax: 86-0371-67967626;

E-mail: [hzy91@163.com](mailto:hzy91@163.com).

**Key words:** GALNT4, ASK1, phosphorylation, dimerization, cardiac hypertrophy,

**Financial support:** This work was supported by The National Natural Science Foundation of China (Grant No.81770047) and the Scientific and Technological Project of Henan Province (202102310364).

**Conflict of interest:** None.

**Supplementary tables**

**Supplementary table 1**

| Anbibody | Manufacturer | Catalogue number | Source of species | Dilution |
| --- | --- | --- | --- | --- |
| GALNT4 | Proteintech | 12897-1-AP | rabbit | 1:1000 |
| p-ASK1 | CST | 3764 | rabbit | 1:1000 |
| ASK1 | Abclonal | A6274 | rabbit | 1:1000 |
| p-ERK | CST | 4370 | rabbit | 1:1000 |
| ERK | CST | 4695 | rabbit | 1:1000 |
| P-JNK | CST | 4668 | rabbit | 1:1000 |
| JNK | CST | 9252 | rabbit | 1:1000 |
| p-p38 | CST | 4511 | rabbit | 1:1000 |
| p38 | CST | 9212 | rabbit | 1:1000 |
| GAPDH | CST | 2118 | rabbit | 1:5000 |
| Bax | CST | 2772 | rabbit | 1:1000 |
| C-Caspase3 | CST | 9664 | rabbit | 1:1000 |
| Flag | MBL | M185-3LL | mouse | 1:1000 |
| Myc | MBL | M047-3 | mouse | 1:1000 |
| HA | MBL | M180-3 | mouse | 1:1000 |

**Supplementary table 2**

| Gene name | Forward primer (mouse) | Reverse primer (mouse) |
| --- | --- | --- |
| *Galnt4* | GCTGGCCCTTTTAACACTGG | CTCTCCCCCATTCCCCAAGA |
| *Anp* | TCGGAGCCTACGAAGATCCA | TTCGGTACCGGAAGCTGTTG |
| *Bnp* | GAAGGACCAAGGCCTCACAA | TTCAGTGCGTTACAGCCCAA |
| *Myh7* | CAACCTGTCCAAGTTCCGCA | TACTCCTCATTCAGGCCCTTG |
| *Collagen Iα* | TGCTAACGTGGTTCGTGACCGT | ACATCTTGAGGTCGCGGCATGT |
| *Collagen III* | ACGTAAGCACTGGTGGACAG | CCGGCTGGAAAGAAGTCTGA |
| *Ctgf* | TGACCCCTGCGACCCACA | TACACCGACCCACCGAAGACACAG |
| *Gapdh* | ACTCCACTCACGGCAAATTC | TCTCCATGGTGGTGAAGACA |
|  |  |  |
| Gene name | Forward primer (rat) | Reverse primer (rat) |
| *Galnt4* | GACAGGCGGACATCAAGAAT | CCCTGAAAGACAGCTCAAGG |
| *Anp* | AAAGCAAACTGAGGGCTCTGCTCG | TTCGGTACCGGAAGCTGTTGCA |
| *Bnp* | TGCCCCAGATGATTCTGCTC | TGTAGGGCCTTGGTCCTTTG |
| *Gapdh* | CAGTGCCAGCCTCGTCTCAT | AGGGGCATCCACAGTCTTC |

| Gene | Primer sequence(5’-3’) | |
| --- | --- | --- |
| AAV9-Galnt4 | F | ACCCCGGTCCGGCTAGCCACCATGGCGGTGAGGTGGACTTG |
|  | R | TCTCCACTGCCGAATTCTTTCTCAAAACTCCAAATTTGATTTTTATC |
| AdGalnt4 | F | GGCTAGCGATATCGGATCCGCCACCATGGCCGTGAGGTGGACCTG |
|  | R | CGTCCTTGTAATCACTAGTTTTCTCAAACCTCCAGAGCTGG |
| AdshGalnt4 | F | CCGGTGAGTGTAACACTGGTTGGTTCTCGAGAACCAACCAGTGTTACACTCATTTTTG |
|  | R | AATTCAAAAATGAGTGTAACACTGGTTGGTTCTCGAGAACCAACCAGTGTTACACTCA |

| Gene name | | Forward primer (mouse) | Reverse primer (mouse) |
| --- | --- | --- | --- |
| *Flag-ASK1(1-678)* | | TCGGGTTTAAACGGATCCATGAGC  ACGGAGGCGGACG | GGGCCCTCTAGACTCGAGTCAATCATATTC  ATAGTCATACTCCAG |
| *HA-ASK1(1-678)* | TCGGGTTTAAACGGATCCATGAGCA  CGGAGGCGGACG | | GGGCCCTCTAGACTCGAGTCAATCATATTC  ATAGTCATACTCCAG |
| *Myc-Galnt4* | TCGGGTTTAAACGGATCCATGGCGGTG  AGGTGGACTTGG | | GGGCCCTCTAGACTCGAGCTATTTCTCAA  AACTCCAAATTTG |
